# In Silico, In Vitro, and In Vivo Antidiabetic Activity of an Alkaloid, 1, 2‐Dimethoxy‐12‐Methyl‐7‐(3‐Methylbut‐2‐en‐1‐yl)‐12, 13‐Dihydro [1,3] Benzodioxolo [5,6‐c] Phenanthridin‐13‐ol, Isolated From a Zimbabwean Herbal Antidiabetic Medicine

**DOI:** 10.1155/bmri/4887174

**Published:** 2026-03-16

**Authors:** Pamhidzai Dzomba, Pardon Mugari, Stephen Nyoni, Elias Mudewairi

**Affiliations:** ^1^ Chemistry Department, Faculty of Science and Engineering, Bindura University of Science Education, Bindura, Zimbabwe, buse.ac.zw; ^2^ Hilbright Science College, Eastlea Campus, Harare, Zimbabwe; ^3^ Chinhoyi University of Technology, Chemistry Department, Chinhoyi, Zimbabwe, cut.ac.zw

**Keywords:** 1 2-dimethoxy-12-methyl-7-(3-methylbut-2-en-1-yl)-12 13-dihydro [13] benzodioxolo [56-c] phenanthridin-13-ol, diabetes mellitus Type 2, molecular docking, protein tyrosine phosphatase 1B

## Abstract

Protein tyrosine phosphatase 1B (PTP1B) is a crucial drug target for treating diabetes mellitus Type 2 (DMT2) because of its link to insulin resistance. Currently there are no approved clinical drugs targeting PTP1B. Therefore, the present study was aimed at investigating the mode of action of an alkaloid, 1, 2‐dimethoxy‐12‐methyl‐7‐(3‐methylbut‐2‐en‐1‐yl)‐12, 13‐dihydro [1,3] benzodioxolo [5,6‐c] phenanthridin‐13‐ol (1, 2 DMMDBP), previously isolated from a popular Zimbabwean antidiabetic herbal medicine. Molecular docking studies using PDB, 2QBP (catalytic site) and IT48 (allosteric site), in vitro PTP1B enzyme inhibition, and in vivo assays using streptozotocin induced diabetic rats were applied to investigate antidiabetic effect. Drug‐like and toxicity properties were evaluated using SwissADME and Protox 3.0 webservers, respectively. Molecular docking results showed that the test compound (1, 2 DMMDBP) has a greater binding affinity (11.1 kcal/mol, rmsd, 0.000 Å) for the allosteric site than the catalytic site (9.6 kcal/mol, rmsd, 0.000 Å). In vitro inhibition assay showed that 1, 2 DMMDBP was more potent (IC50 = 1.10 *μ* *M*) than that of ursolic acid (IC50 = 7.13 *μ* *M*). Additionally, in in vivo studies, 1, 2 DMMDBP maintained normal hypoglycemia and mass better than the reference drug metformin. In absorption, distribution, metabolism, and excretion predictive studies, 1, 2 DMMDBP showed good drug‐like properties. It did not violate any of Lipinski′s classic rules. It showed good physicochemical properties such as absorption, (log of skin permeability (log Kp) value was −4.95), bioavailability with a score of 0.55 and biotransformation by cytochrome‐P enzymes CYP1A2 and CYP3A4. Protox 3.0 webserver predicted LD_50_ value of 1000 mg/kg, showing that it may be toxic if swallowed. Based on the evidence presented, 1, 2 DMMDBP is a highly promising compound in the development of potent and selective allosteric modulator drugs of PTP1B for the treatment of DMT2 upon further studies.

## 1. Introduction

Diabetes mellitus Type 2 (DMT2), a condition of hyperglycemia, is becoming a huge problem every day [1]. DMT2 occurs when the body fails to utilize circulating insulin efficiently due to insulin resistance [2]. Insulin resistance occurs when insulin receptor Site 1 (IRS‐1) and insulin receptor Site 2 (IRS‐2) are dephosphorylated. This, in turn, results in a disturbed insulin transduction [3]. Acute DMT2 results in various complications including neuropathy, renopathy, kidney failure, and death [4]. Recently, oxidative stress, proinflammation cytokines, inflammation, and free fatty acids have been reported to be the major mediators of insulin resistance [1]. Inflammation and free fatty acid insulin resistance mediated mechanisms and oxidative stress all converge to afford a potentially unifying mechanism of insulin resistance [5]. In previous insulin‐resistant studies, insulin receptors were found to be dephosphorylated in both rats′ models and human skeletal muscles [6]. Dephosphorylation disturbed insulin receptors and substrates from binding in disruptive yeast trihybrid experiments [7]. Also, insulin receptors were dephosphorylated in human cells under conditions of insulin resistance [8]. Thus, drugs that stop dephosphorylation of insulin receptors should be sought to reverse insulin resistance. Such drugs should target protein tyrosine phosphatase 1B (PTP1B), which has negative regulation by removing phosphate groups from the insulin receptor activation segment. Inhibiting the PTP1B negative regulatory function on insulin signaling allows the insulin receptor to remain phosphorylated and activated. Thus, enhancing cells′ response to insulin.

PTP1B belongs to the protein tyrosine phosphatase family of enzymes that plays a crucial role in the physical pathogenesis of DMT2 [6]. PTP1B consists of two different portions, the catalytic site and the allosteric site, both of which are important components for enzyme activity and control [9]. The core domain of the enzyme consists of the active site that dephosphorylates tyrosine residues on target proteins (insulin receptor site) [10]. The allosteric site is found on the C‐terminal domain, consisting of 20 residues from the catalytic site [11]. The allosteric site is less polar and poorly conserved among both PTP1B and other cellular PTPs [12]. Therefore, finding natural drug candidates that can interact more with the allosteric site presents an attractive strategy for creating PTP1B inhibitors that have good bioavailability and selectivity.

In our previous study, [13] an alkaloid 1, 2‐dimethoxy‐12‐methyl‐7‐(3‐methylbut‐2‐en‐1‐yl)‐12, 13‐dihydro [1,3] benzodioxolo [5,6‐c]phenanthridin‐13‐ol (1, 2 DMMDBP) was isolated from a popular Zimbabwean antidiabetic herbal medicine. Although the chemical structure of the isolated alkaloid has been reported before [13], the mode of action and drug‐like properties of the isolated compound remain unknown. To investigate the efficacy, mode of action and drug‐like properties of the alkaloid, in silico, in vitro, and in vivo analysis methods were utilized. Therefore, the present study demonstrates for the first time the allosteric PTP1B modulation and drug‐like properties of the compound.

## 2. Materials and Methods

### 2.1. In Silico Assay

To investigate the interaction and binding of the new alkaloid, 1, 2‐dimethoxy‐12‐methyl‐7‐(3‐methylbut‐2‐en‐1‐yl)‐12, 13‐dihydro [1,3] benzodioxolo [5,6‐c]phenanthridin‐13‐ol with PTP1B, molecular docking experiments were carried out with AutoDock 4.2 software [2]. The x‐ray crystallographic structures of PTP1B, 2QBP (catalytic site) and IT48 (allosteric site) were obtained from the RCSB Protein Data Bank website [14, 15]. The selective catalytic inhibitor: 5‐(3‐{[1‐(benzylsulfonyl)piperidin‐4‐yl]amino}phenyl)‐4‐bromo‐3‐(carboxymethoxy)thiophene‐2‐carboxylic acid and the selective allosteric inhibitor, 3‐(3,5‐dibromo‐4‐hydroxy‐benzoyl)‐2‐ethyl‐benzofuran‐6‐sulfonic acid (4‐sulfamoyl‐phenyl)‐amide were used to locate the respective binding site of the protein. Energy minimization was performed using the YASARA force field minimization server. Protein preparation was conducted using Discovery Studio 2025 BIOVIA software (Accelrys, Inc., San Diego, California, United States). The 3D structures of 2‐dimethoxy‐12‐methyl‐7‐(3‐methylbut‐2‐en‐1‐yl)‐12, 13‐dihydro [1,3] benzodioxolo [5,6‐c] phenanthridin‐13‐ol were prepared and minimized using Chemdraw and PYMOL software. Docking simulations were performed using AutoDock tools to assess the appropriate binding poses of the ligand molecules with different protein structures. For docking calculations, polar charges were added by default, the rotatable bonds were set by the AutoDock tools, and all torsions were allowed to rotate. The grid maps were generated by the AutoGrid program, and the grid box size of 30 × 30 × 30 was used. The binding aspect of PTP1B residues and their corresponding binding affinity score were regarded as the best molecular interaction. The results were visualized and analyzed using Discovery Studio 2025 software.

### 2.2. Absorption, Distribution, Metabolism, and Excretion (ADMET) Predictions

The test compound, 1, 2 DMMDBP, was screened based on Lipinski′s rule of five [16]. SwissADME [17] was utilized for the in silico physicochemical properties assessment. The test compound acute oral toxicity prediction was performed by Protox 3.0 webserver [18].

### 2.3. In Vitro PTP1B Inhibition Assay

The in vitro enzyme inhibition assessment was performed using Sánchez Calero et al., [19]s′ protocol with slight changes in concentration ranges used. The substrate, p‐nitrophenyl phosphate (pNPP) acquired from Merck, South Africa, was used to measure enzyme activity. A buffer comprised of 25‐mM Tris–HCl (pH 7.5), 2‐mM b‐mercaptoethanol, 1 mM (ethylenediaminetetraacetic acid), and 1‐mM dithiothreitol was utilized. Test inhibitor 10 *μ*L of 0.1–10‐*μ*M concentration range was added to 20 *μ*L of enzyme (1 *μ*g/mL), and then the contents were mixed with 40 *μ*L of 4‐mM pNPP in 130 *μ*L of the given buffer using a 96 well plate incubated at 37°C for 10 min. The pNP produced as a result of pNPP dephosphorylation was monitored spectrophotometrically at 405 nm for 30 min. The positive reference inhibitor was ursolic acid. The IC_50_, the concentration of the compound that inhibited 50% of enzyme activity, was determined graphically. The percent inhibition was also calculated using the equation, *%*inhibition = (rate of control reaction (without inhibitor) − rate ofsample reaction)/rate of control reaction × 100. The experiments were performed in triplicate. The nonenzymatic hydrolysis of the substrate was corrected by measuring the absorbance of the negative control with no PTP1B enzyme.

### 2.4. In Vivo Assay

In vivo studies were performed using healthy male Wister rats 200–250 g [20]. Antidiabetic activity was investigated by inducing diabetes in the rats by administering streptozotocin in conjunction with a high‐fat diet (olive oil). The rats were housed in polypropylene cages with floors lined with husks. Standard conditions of temperature 25 ± 2°C, 12:12 light to dark cycle. The rats were fed with standard pellets twice per day and had free access to clean water. All experiments were conducted in compliance with international laws and institutional animal ethics guidelines. Healthy animals were fasted overnight, and then, diabetes was induced by administering streptozotocin 60 mg/kg dissolved in 0.1‐M citrate buffer, pH 4.5 by an intraperitoneal injection [21]. Treatment with 1, 2 DMMDBP was done by an intraperitoneal injection of the compound, using the same concentration as metformin (40 mg/kg in saline solution) every morning for 40 days. Control animals received the buffer only. Body masses of animals were monitored continuously. Diabetes was determined by measuring the rats′ blood glucose using a tail cut after 3 days of STZ injection using glucometer (Accu‐Check, Roche Diagnostics, Basel). Rats found with blood glucose levels of greater than 200 mg/dL were considered to be diabetic and utilized in the experiment [22]. The rats, 10 in each group, were randomly divided into four groups and received treatment as follows:

Group 1: normal rats, control + buffer and saline only.

Group 2: diabetic, STZ‐induced rats treated with 40 mg/kg/day 1, 2 DMMDBP.

Group 3: diabetic, STZ‐induced rats treated with 40 mg/kg/day metformin.

Group 4: diabetic, STZ‐induced rats + buffer and saline only.

### 2.5. Statistical Analysis

Results for PTP 1B/TCPTP inhibition assay and in vivo analysis are expressed as mean ± standard deviation of triplicate measurements. Using IBM SPSS Statistics 26, statistical comparison was carried out by ANOVA analysis and Tukey post hoc test at 0.05 significance level.

## 3. Results

### 3.1. Molecular Docking Studies of PTP1B Inhibition

**Figure 1:**
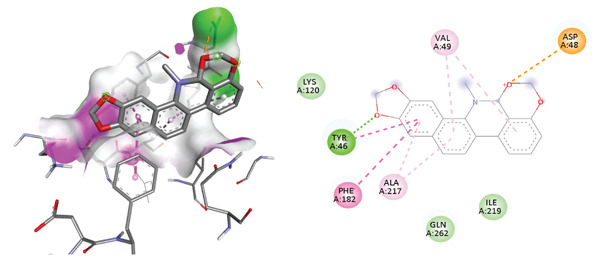
Inhibition mode of 1, 2‐dimethoxy‐12‐methyl‐7‐(3‐methylbut‐2‐en‐1‐yl)‐12, 13‐dihydro [1,3] benzodioxolo [5,6‐c] phenanthridin‐13‐ol for PTP1B and 2D ligand interaction at the catalytic site. Dashed lines show the interacting bonds, green hydrogen bonding, orange.electrostatic interactions, and dark–light pink hydrophobic interactions.

**Figure 2:**
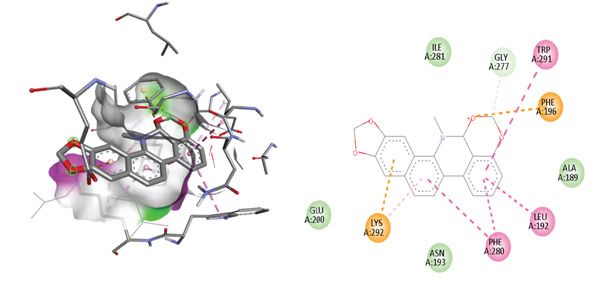
Inhibition mode of 1, 2‐dimethoxy‐12‐methyl‐7‐(3‐methylbut‐2‐en‐1‐yl)‐12, 13‐dihydro [1,3] benzodioxolo [5,6‐c] phenanthridin‐13‐ol for PTP1B and 2D ligand interaction at the allosteric site. Dashed lines showing the interacting bonds, green hydrogen bonding, orange electrostatic interactions, and dark–light pink hydrophobic interactions.

To investigate the ability of the compound 1, 2 DMMDBP to inhibit PTP1B enzyme by interacting at the active and allosteric sites, molecular docking studies using Autodock 4.2 were carried out. To validate and optimize the docking method, a selective catalytic inhibitor: 5‐(3‐{[1‐(benzylsulfonyl)piperidin‐4‐yl]amino}phenyl)‐4‐bromo‐3‐(carboxymethoxy)thiophene‐2‐carboxylic acid and the selective allosteric inhibitor A: 3‐(3,5‐dibromo‐4‐hydroxy‐benzoyl)‐2‐ethyl‐benzofuran‐6‐sulfonic acid (4‐sulfamoyl‐phenyl)‐amide were re‐docked (14,15). The root mean square deviation (RMSD) values were then determined by comparing the best pose generated using the docking protocol with the selective catalytic and allosteric inhibitors in Autodock Vina 4.2. The validated method was then used to investigate the binding pose of the compound 1, 2 DMMDBP. The predicted binding modes of 1, 2 DMMDBP on the catalytic site are shown in Figures 1 and 2. As shown in Figure 1 and Table 1 the oxygen atom on the dioxane ring of 1, 2 DMMDBP form hydrogen bond interaction with Tyr 46 residue and the Ph–OH group, an electrostatic interaction with Asp 48 residues with binding energy of −9.6 kcal/mol, RMSD = 0.000 *Å*, indicating a perfect identical match. Furthermore, the residue Tyr 46 was observed to be involved in *π*–*π* stacked hydrophobic interactions, whereas Phe 182 showed *π*–*π* T‐shaped, and Val 49 and Ala217 showed *π*‐alkyl hydrophobic interactions. At the catalytic site, the 1, 2 DMMDBP only bonded with one important catalytic residue Tyr46 and not with the other important catalytic residues, Cys215 and Gln262. Cys 215 is the key active site residue in the activity of PTP1B [23]. This cysteine residue is highly conserved among PTP family members. For the reference ligand oxygens on −COOH groups were found to be responsible for hydrogen bond interactions with the important catalytic residues Tyr46, Cys215, and Gln262 [2].

**Table 1:** Binding site residues and docking scores of the ligand and PTP1B obtained using AutoDock 4.2.

Site	Binding energy (kcal/mol)	Hydrogen bond interaction	Electrostatic interaction	Hydrophobic interactions
Catalytic site	−9.6	Tyr46	Asp48	Val49Tyr46Phe182Ala217
Allosteric site	−11.1	Gly277	Phe196Lys292	Phe280Leu192Trp291Lys292

Molecular docking on the allosteric pocket results are shown in Figure 2 and Table 1. To compare the interaction between 1, 2 DMMDBP and PTP1B at allosteric site, co‐ligand in PTP1B (1T48), 3‐(3,5‐dibromo‐4‐hydroxy‐benzoyl)‐2‐ethyl‐benzofuran‐6‐sulfonic acid (4‐sulfamoyl‐phenyl)‐amide, was docked. The reference ligand showed one hydrogen bond with Asn 193 and four hydrophobic interactions at Lys 292, Glu276, Trp291 Phe 280, Leu192, and Phe 196. The test compound, 1, 2 DMMDBP showed strong similar interactions and orientation with the important allosteric residues including, Phe280, Phe 196, Leu192, Trp291 via *π*–*π* stacked hydrophobic interactions, Lys 292 via electrostatic interactions and Gly 277 via hydrogen bonding with a high binding affinity of ‐11.1 kcal/mol, RMSD = 0.000 Angstrom. As compared with the catalytic site, the test compound forms a tighter binding at the allosteric pocket than at the catalytic site (9.6 kcal/mol). Also the test compound is a more potent allosteric binder than the reference binder (7.2 kcal/mol).

### 3.2. ADMET Prediction

The drug‐like potential results of the test compound, as evaluated using the “Lipinski′s rule of five”, are shown in Table 2. The molecular weight is smaller than 500 Da. The number of hydrogen bond donors and hydrogen bond acceptors are all smaller than 5 and 10, respectively. Log *p* value is also smaller than five. Therefore, the test compound does not violate any of the classic Lipinski rules. This indicates favorable druglike properties of the test compound. The compound′s rotatable bonds and total polar surface area values fit within the favorable ranges, therefore suggesting good oral bioavailability and intestinal absorption. The log *S* value was predicted as −6.17, suggesting that it has good solubility. The predicted physicochemical properties are shown in Table 2. The predicted ADME parameters are shown in Table 3. The log of skin permeability (log Kp) value was −4.95. Thus, the compound was predicted to be absorbed well in the gastrointestinal and the permeating barrier. The bioavailability score of 0.55 shows that the compound will be absorbed and effectively move around in the bloodstream after oral administration, and the synthetic accessibility of 4.29 shows that the compound is a candidate that can reasonably be synthesized. Several cytochrome P enzymes play a vital role in drug biotransformation, including CYP1A2, CYP2C19, CYP2C9, CYP2D6, and CYP3A4. The test compound was predicted to inhibit CYP2C19, CYP2C9, and CYP2D6, but does not inhibit CYP1A2 and CYP3A4. It was also shown to be a substrate of P‐glycoprotein (P‐gp). Protox 3.0 webserver was used to predict the LD_50_ value of the test compound, which was found to be 1000 mg/kg. Based on this predicted LD_50_ value, the test compound was classified as a drug that may be toxic if swallowed.

**Table 2:** Physicochemical properties of the 1, 2 DMMDBP.

Property	Value
MW g/mol	433.5
Log P	3.52
nHBD	1
nHBA	5
TPSA	60.39
MR	128.5
Lipinski violation	0
Log S	‐6.17
nRootB	4

Abbreviations: log P, log of octanol/water partition coefficient; log S, log of solubility; MR, molar refractivity; MW, molecular weight; nHBA, number of hydrogen bond acceptor(s); nHBD, number of hydrogen bond donor(s); nRootB, number of rotatable bond(s); TPSA, total polar surface area.

**Table 3:** ADME predicted results.

Predicted	Score
log Kp (cm/s)	−4.95
GI absorbance	High
BBB per	Yes
P‐gp substrate	Yes
CYP1A2 inhibitor	No
CYP2C19 inhibitor	Yes
CYP2C9 inhibitor	Yes
CYP2D6 inhibitor	Yes
CYP3A4 inhibitor	No
Bioavailability	0.55
Synthetic accessibility	4.29

Abbreviations: ADME, absorption, distribution, metabolism, and excretion; BBB Per, blood–brain barrier permeability; CYP, cytochrome‐P; GI Abs, gastrointestinal absorption; Log Kp, log of skin permeability; P‐gp, P‐glycoprotein.

### 3.3. In Vitro PTP1B Inhibition Assay

PTP1B in vitro inhibitory capacity of 1, 2 DMMDBP was evaluated using p‐NPP as a substrate, and the results were expressed as IC_50_ values, which were determined by linear regression analysis (Table 4). The compound showed strong inhibitory activity on PTP1B with percent inhibition of 88 ± 2.33 and IC_50_ value of 1.10 *μ*M, which was higher than that of standard ursolic acid with percent inhibition of 66 ± 3.62 and IC_50_ = 7.13 ± 0.15 *μ* *M*. This significant in vitro activity supports the high binding affinity 11.1 kcal/mol observed at the allosteric site in molecular docking studies.

**Table 4:** In vitro PTP inhibitory activity.

Compound	IC50, *μ*M	% PTP 1B inhibition
Test inhibitor	1.10 ± 0.71	88 ± 2.33
Ursolic acid	7.13 ± 0.15	66 ± 3.62

### 3.4. In Vivo Antidiabetic Activity of the 1, 2 DMMDBP

#### 3.4.1. Effect of 1, 2 DMMDBP on Blood Glucose Level in STZ‐Induced Diabetic Rats

**Figure 3:**
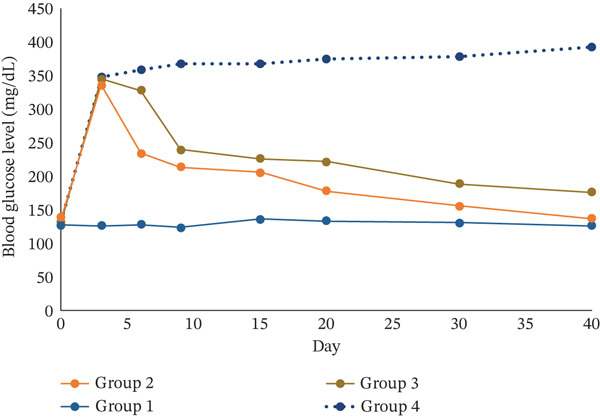
Antidiabetic effect of 1, 2 DMMDBP on STZ‐induced diabetic rats. Group 1: Normal rats, control + buffer and saline only; Group 2: Diabetic, STZ‐induced rats treated with 40 mg/kg/day 1, 2 DMMDBP; Group 3: Diabetic, STZ‐induced rats treated with 40 mg/kg/day metformin; and Group 4: Diabetic, STZ‐induced rats + buffer and saline only.

The effect of repeated administration of 1, 2 DMMDBP on blood glucose level of rats is shown in Table 5. Intraperitoneal injection at a dosage of 60 mg/kg streptozotocin induced diabetes in most of the rats with a success rate of 85%. Levels of blood glucose in rats increased after 3 days, and thereafter, it became almost stable in diabetic rats with no treatment (Group 4), Figure 3, and the levels remained high for 40 days. The blood glucose remained significantly above normal, ranging from 347 to 392 mg/dL. The blood glucose of the diabetic mice administered the test compound Table 5 and Figure 3 quickly decreased to < 240 mg/dL 10 days after, with a sharp increase at Day 3. After 10 days, the levels decreased steadily to < 200 mg/dL at Day 40. The pattern was not significantly different from the blood group of diabetic rats treated with 40 mg/kg/day metformin (Group 3) one way ANOVA, Tukey post hoc test, *p* > 0.05. State of hypoglycemia was reached at Day 25, and at Day 40, there was no statistical difference in blood glucose between the diabetic rats (Group 2) and the normal group, Group 1 *p* > 0.05. The normal group treated with buffer only (Group 1) showed normal blood glucose levels ranging from 124.12–135.14 mg/dL, and there was no statistical difference between the blood glucose levels for Days 1 and 40. The blood glucose of the diabetic rats was statistically different on Days 1 and 3, one‐way ANOVA, Tukey post hoc test *p* < 0.05 and the value of above 200 mg/dL showed a state of hyperglycemia. The state of hypoglycemia for the diabetic rats was statistically not different at Days 40 and 0, *p* > 0.05, before the injection of STZ, showing that the test compound was very effective in normalizing the blood sugar levels.

**Table 5:** Blood glucose levels in mg/dL for the evaluation of the antidiabetic effect of 1, 2 DMMDBP.

Group	*N*	Before injection	After STZ injection, day						
			3	6	9	15	20	30	40
1	10	135.14 ± 3.66	124.12 ± 5.26	134.16 ± 3.73	133.67 ± 6.77	126.12 ± 3.11	131.62 ± 5.80	133.11 ± 5.77	133.14 ± 3.22
2	10	139.12 ± 6.72	336.43 ± 9.13	234.34 ± 6.63	213.93 ± 9.22	206.12 ± 3.95	178.02 ± 9.71	156.34 ± 5.77	137.51 ± 6.43
3	10	132.62 ± 5.71	344.67 ± 8.06	328.33 ± 3.06	239.87 ± 9.11	226.45 ± 7.67	221.73 ± 6.98	188.91 ± 6.23	176.91 ± 9.42
4	10	132.11 ± 2.43	367.43 ± 9.12	369.34 ± 6.71	366.97 ± 7.17	368.44 ± 5.41	374.82 ± 8.91	378.66 ± 4.64	392.51 ± 3.89

*Note:* Group 1: Normal rats, control + buffer and saline only; Group 2: Diabetic, STZ‐induced rats treated with 40 mg/kg/day 1, 2 DMMDBP; Group 3: Diabetic, STZ‐induced rats treated with 40 mg/kg/day metformin; and Group 4: Diabetic, STZ‐induced rats + buffer and saline only.

#### 3.4.2. Effect of the 1, 2 DMMDBP on Body Mass in STZ‐Induced Diabetic Rates

The masses of the rats are shown in Table 6. Body mass is one of the most important parameters to evaluate in antidiabetic studies. The body mass of the untreated diabetic rats, Group 4, changed significantly from Day 0 to Day 40, with the highest negative mass change of −11.69, showing breakdown of muscle and fats due to lack of insulin action. There was a significant difference *p* > 0.05 between the masses of diabetic rats administered the test compound and those on metformin, showing that the test compound maintained the body mass of the rats much better than metformin. There was no significant difference between the body mass of the diabetic rats administered 1, 2 DMMDBP and those of the normal group, with a positive mass change of 0.07 compared with 0.79, respectively.

**Table 6:** Body masses of rats during the experiment.

Group	*N*	Before injection	Day							
			3	6	9	15	20	30	40	Mass change
1	10	220.81 ± 0.76	224.66 ± 0.16	222.07 ± 0.43	223.67 ± 0.27	226.33 ± 0.98	221.44 ± 0.80	222.67 ± 0.85	221.60 ± 0.22	0.79
2	10	210.53 ± 0.86	210.43 ± 0.52	210.52 ± 0.45	211.02 ± 0.66	210.63 ± 0.53	210.42 ± 0.20	210.57 ± 0.67	210.60 ± 0.31	0.07
3	10	221.67 ± 0.73	223.39 ± 0.19	222.43 ± 0.67	223.23 ± 0.96	222.62 ± 0.77	222.67 ± 0.09	222.12 ± 0.73	220.10 ± 0.54	−1.67
4	10	250.02 ± 0.88	246.22 ± 0.32	243.16 ± 0.51	236.67 ± 0.72	236.12 ± 1.31	232.01 ± 1.22	233.54 ± 0.92	238.33 ± 0.37	−11.69

*Note:* Group 1: Normal rats, control + buffer and saline only; Group 2: Diabetic, STZ‐induced rats treated with 40 mg/kg/day 1, 2 DMMDBP; Group 3: Diabetic, STZ‐induced rats treated with 40 mg/kg/day metformin; and Group 4: Diabetic, STZ‐induced rats + buffer and saline only.

## 4. Discussion

Cases of DMT2 are increasing in an exponential pattern and are now believed to be the leading cause of death, particularly in developing countries with economies transitioning to growth [24]. PTP1B is a major player in the physiopathology of DMT2 [6]. PTP1B dephosphorylates the insulin receptor and insulin receptor substrate proteins, thereby negatively regulating insulin signaling and promoting resistance [25]. In addition, PTP1B plays a significant role in leptin signaling by dephosphorylation of the downstream effector of the leptin receptor Janus kinase 2 (JAK 2) [26]. Because PTP1B is linked to the development of insulin resistance, it is an appropriate drug target for discovering PTP 1B modulating inhibitors for DMT2 [2]. To date, only a few compounds have moved to clinical trials, including JTT‐551 (a mixed type inhibitor), trodusquemine (an allosteric inhibitor), and ertiprotafib (a noncompetitive inhibitor), and some recently discovered inhibitors [27]. Unfortunately, none of them have passed clinical trial stages because they were discontinued due to lack of specificity, serious side effects, and lack of adequate efficacy [8]. Thus, it is essential to continue to research new selective and effective PTP1B modulators for DMT2.

Targeting PTP1B presents challenges because PTPs are hugely conserved and have a polar catalytic site [26]. Polar modulators exhibit poor bioaccessibility. The catalytic domain of T‐cell PTPs, essential for various cellular activities, shares 72% sequence identity and 86% similarity with the catalytic domain of PTP 1B; therefore, it presents great constraints in the development of effective and PTP1B‐selective inhibitors. Liu et al. [8] proposed three ways of developing specific and effective PTP1B modulator compounds: (i) developing dual inhibitors that bind to the active site and to another secondary site that is specific only to PTP1Bs. (ii) developing compounds that target the allosteric pockets of PTP1Bs. The allosteric site is significantly less polar and is poorly conserved among PTPs [28]. This means that successfully achieving PTP selectivity and higher bioavailability targeting the allosteric pocket is a noble strategy. Accordingly, in an effort to search for a potent and selective PTP1B inhibitor, we investigated in silico, in vitro, and in vivo antidiabetic activity of 1, 2 DMMDBP isolated from a Zimbabwean traditional antidiabetic medicine.

The tested compound 1, 2 DMMDBP exhibited interesting interaction with PTP1B. Comparing docking at the catalytic and allosteric sites, 1, 2 DMMDBP showed greater binding affinity (−11.1 kcal/mol) for the allosteric site than the catalytic site (−9.6 kcal/mol). It also bonded with five important allosteric pocket residues, Phe280, Phe 196, Leu192, Trp291, and Lys 292 [29] than only one important catalytic site residue, Tyr46. The test compound 1, 2 DMMDBP did not bind with the very important and highly conserved PTPs residue Cys 215 [30]. This shows that 1, 2 DMMDBP is a potentially highly potent and allosteric PTP1B modulator drug. It is less likely to affect the other cellular PTPs. In in vitro studies, 1, 2 DMMDBP showed very high inhibitory activity with an IC_50_ of 1.10 *μ*M, which is much lower than that of ursolic acid, 7.13 *μ*M, showing that it is more potent than ursolic acid. Interestingly, the most potent reference PTP 1B inhibitors, sodium orthovanadate and 2‐[(Carboxycarbonyl)amino]‐4,5,6,7‐tetrahydrothieno[2,3‐c]pyridine‐3‐carboxylic acid hydrochloride (TCS 401), were reported to have IC_50_ values of 0.12 and 0.18 *μ*M, respectively [31]. This indicates that 1, 2 DMMDBP is a highly potent PTP1B modulator candidate, with an IC50 value close to that of the reference standards, sodium orthovanadate, and TCS 401, as reported values [31].

The in vivo results in Table 5 and Figure 3 show that 1, 2 DMMDBP reduced the blood glucose of diabetic rats in the same pattern as metformin; however, it was more potent than metformin (profile of 1, 2 DMMDBP lower than for metformin) Figure 3. The test compound 1, 2 DMMDBP also showed that it did not cause significant changes in mass. In in silico ADME predictions, 1, 2 DMMDBP was predicted to be well‐absorbed in the gastrointestinal tract, and it permeates the blood–brain barrier. It can also be biotransformed in the body by CYP1A2 and CYP3A4 enzymes. It was also predicted to have an LD_50_ = 1000 mg/kg, showing that it may require no or minor modifications.

## 5. Conclusion

Overall, this study reports the first in silico, in vitro, and in vivo investigation of the PTP1B inhibitory activity and antihyperglycemic effect of the alkaloid 1, 2‐dimethoxy‐12‐methyl‐7‐(3‐methylbut‐2‐en‐1‐yl)‐12, 13‐dihydro [1,3] benzodioxolo [5,6‐c] phenanthridin‐13‐ol isolated from a popular local antidiabetic herbal drug. The present results show that 1, 2‐dimethoxy‐12‐methyl‐7‐(3‐methylbut‐2‐en‐1‐yl)‐12, 13‐dihydro [1,3] benzodioxolo [5,6‐c] phenanthridin‐13‐ol demonstrated greater binding affinity at the PTP1B allosteric site and potent in vitro PTP1B inhibitory activity and in vivo antihyperglycemic effect on diabetes rats. It also showed favorable drug‐like properties in ADME studies. Based on the evidence presented in the present study, 1, 2‐dimethoxy‐12‐methyl‐7‐(3‐methylbut‐2‐en‐1‐yl)‐12, 13‐dihydro [1,3] benzodioxolo [5,6‐c] phenanthridin‐13‐ol is a promising candidate for developing a highly potent and selective allosteric inhibitor of PTP1B for the treatment of DMT2 upon further studies.

## Author Contributions

All authors conceptualized the study. Pamhidzai Dzomba and Pardon Mugari carried out laboratory experiments. Pamhidzai Dzomba carried out the in silico studies. Stephen Nyoni managed the literature. Stephen Nyoni and Pamhidzai Dzomba wrote the first draft. Pamhidzai Dzomba wrote the final draft.

## Funding

This study was supported by Hilbright Science Education.

## Disclosure

All authors reviewed and approved the final draft and accepted responsibility for the entire content of this manuscript and approved its submission.

## Ethics Statement

All in vivo experiments were conducted in compliance with international laws, Lab Anim. Sci. 43:535–540, and institutional animal ethics guidelines and approval.

## Conflicts of Interest

The authors declare no conflicts of interest.

## Data Availability

The data that support the findings of this study are available on request from the corresponding author. The data are not publicly available due to privacy or ethical restrictions.
